# Reproductive Technologies Used in Male Neo-Tropical Hystricomorphic Rodents

**DOI:** 10.3390/ani12010034

**Published:** 2021-12-24

**Authors:** Kavita Ranjeeta Lall, Kegan Romelle Jones, Gary Wayne Garcia

**Affiliations:** 1 Department of Food Production (DFP), Faculty of Food and Agriculture (FFA), University of the West Indies (UWI), St. Augustine Campus, St. Augustine 999183, Trinidad and Tobago; k_lee_24@yahoo.com (K.R.L.); prof.gary.garcia@gmail.com (G.W.G.); 2 Department of Basic Veterinary Sciences (DBVS), School of Veterinary Medicine (SVM), Faculty of Medical Sciences (FMS), University of the West Indies (UWI), St. Augustine Campus, St. Augustine 999183, Trinidad and Tobago

**Keywords:** agouti, *Dasyprocta leporina*, lappe, *Agouti paca*, capybara, *Hydrochoerus hydrochaeris*, reproductive tract, conservation, domestication

## Abstract

**Simple Summary:**

This paper is a literature review on the reproductive technologies used in male Neo-tropical hystricomorphic rodents. It is the first of two literature reviews to be completed in order to aid future experiments on the estrus synchronization and artificial insemination of the agouti (*Dasyprocta leporina*). To improve efficient reproduction, reproductive technologies are commonly used in domesticated animals; therefore, it is wise to explore the feasibility of their application on Neo-tropical rodents, an alternative meat source with the potential to aid in conservation and wildlife farming. However, there must first be a proper understanding of their reproductive anatomy, before reproductive technologies can be utilized. The penis of the agouti and the paca (*Cuniculus paca*), for example, were found to be covered in penile spines with two keratinized spines and two lateral penile cartilages on either side of the glans penis, unlike the capybara (*Hydrochoerus hydrochaeris*). Different methods can be used to collect semen from these animals and, based on the performed review, it has been shown that coconut water powder (ACP-123) is a good diluent for both the agouti and the paca, giving higher spermatozoa parameters compared with those obtained via electro ejaculation.

**Abstract:**

This review, which is the first of two, focuses on the male reproductive anatomy and reproductive technologies used in Neo-tropical hystricomorphic rodents with the potential for domestication, which are the agouti (*Dasyprocta leporina*), the capybara (*Hydrochoerus hydrochaeris*) and the paca (*Cuniculus paca*). We consider over seventy references spanning from 1965 to 2020, with the majority of work being done in the past twenty years. Knowledge of the reproductive tract and reproductive technologies is critical to the conservation and preservation of these species. Although all three animals had similarities in their anatomy, such as no overt scrotums and testes located intra-abdominally in the inguinal region, some had unique features—for example, the agouti and the paca had penile spines, and two lateral penile cartilages. High spermatogenic efficiency was noted in the agouti and the paca, making them good candidates for increasing their reproductive performance in conservation programs. A review of the literature has shown that there is increasing work taking place on the reproductive technologies used in these animals; however, a lot of work is still lacking, as, to the author’s knowledge, standard protocols and artificial insemination procedures are yet to be established.

## 1. Introduction

Hystricomorphic rodents belong to the suborder of Hystricomorpha, which is a sub-division of the Order Rodentia [1]. According to Wood [2], hystricomorphy is one of four types of rodent skull, differentiated based on the zygomasseteric system; the other three types are protrogomorphy, sciuromorphy and myomorphy. The anterior section of the masseter medialis of hystricomorphic rodents runs from the medial side of the orbit, through an enlarged infraorbital foramen, to the lateral surface of the rostrum. In extreme cases, such as in the *Hydrochoerus* spp., its origin extends as far forward as the pre-maxilla. As a result, this gives an almost horizontal movement upon contraction of this muscle, influencing the horizontal action of the masseter superficialis.

Neo-tropical hystricomorphic rodents with the potential for domestication are important, as they serve as game species [3]. Moreover, also of importance is the ecological role they play, especially the agouti, which is a known scatter-hoarder, the act of which enables a constant supply of food [3,4,5,6,7]. Wildlife farming is therefore important to these animals, as it helps to create a captive bred stock, provides a gene pool for future work, aids in food production, creates employment and development, and helps with conservation [3,8].

Reproductive technologies modify reproductive performances in such a way so as to improve reproduction in animals [9]. The implementation of reproductive technologies or assisted reproductive techniques (such as artificial insemination, in vitro fertilization, gamete cryopreservation, embryo transfer and genetic resource banks) can be used to enhance and optimize breeding programs [10,11,12]. Improving reproduction within breeding programs is important in ensuring the survival of animals such as the *Dasyprocta* spp. (for conservation), as well as to meet the demands for providing an alternative protein source for human consumption, as pollution and other human factors threaten the future of this species [3]. 

Knowledge of the basic anatomy and physiology of the reproductive tract is important before attempting to understand and use assisted reproductive techniques [11]. The *Dasyprocta* spp., pose a problem in this regard, since little is known about the morpho-physiological characteristics of the male and female agouti. This knowledge will be required for application in sustainable production systems, as well as for the development and improvement of protocols [13].

The objectives of this review were to document the gross anatomy and histology of the reproductive systems and reproductive technologies reported for the male agouti (*D. leporina*), capybara (*H. hydrochaeris*) and paca (*C. paca*). This knowledge is important to understand any unique features of the reproductive tract, in order to be able to successfully utilize reproductive technologies, as these animals have a multi-functional role, especially in serving as alternative animal protein sources. Thus, ensuring the continuation of these species is important. 

## 2. Methodology

An extensive literature search was done using several search engines, such as Google Scholar, Pubmed and UWIlinC. To obtain pertinent information, examples of keywords searched included *Dasyprocta leporina, Hydrochoerus hydrochaeris, Cuniculus paca,* male reproduction, male reproductive tract, reproductive technology, artificial insemination and hystricomorphic rodents. The number of articles shortlisted from the search was 300, with only 73 used in the paper due to relevance, spanning the years 1965–2020. The inclusion criteria included the definition of hystricomorphic rodents, anatomy and histology of the male reproductive tract for each species, and reproductive technologies such as electro ejaculation and the collection and preservation of semen. 

## 3. Agouti (*Dasyprocta leporina*)

### 3.1. Gross Anatomyof the Male Reproductive Tract

Testicular growth was slow from 0 to 8 months old, with a sharp increase occurring from 9 months, and testicular biometric parameters being highly correlated to age and body weight of the animals [14]. A pair of oval, pinkish-brown testes were located within scrotal pouches intra-abdominally [15]. Externally, the testes were seen as two subcutaneous lumps on either side of the inner hind legs. The mean testicular volume was 5.39 ± 0.32 cm^3^, while the gonadosomatic index was 0.48 ± 0.02% [15]. Some authors found that the mean testis weight for *D. leporina* was 4.1 ± 0.6 g [16]. Table 1 lists the mean measurements of the testes. Menezes et al. [17] further stated that the external part of the genitals was hidden ventrally within a skin sac, and the scrotal sacs were not clearly demarcated.

The epididymis, which ran the entire length of the testis, had a coiled head enclosed by fat, with an increase in coiling of the body towards the cauda epididymis. The paired vas deferens were creamish-white in color and thick-walled, and terminated in the urethra. The diameter of the vas deferens expanded to form the ampulla (mean diameter 0.25 cm) close to the urethral end [15].

The accessory sex glands of the agouti included the seminal vesicles, the coagulating glands, the prostate gland and the bulbourethral glands [15,17]. The seminal vesicles, which were branched, tightly coiled and pale yellowish-cream in color, were found to be the largest of the accessory sex glands. They were located at the dorsal end of the vas deferens and attached via a short stem, which continued into a main branch from which a number of lobes originated [15,18]. The coagulating glands, found on either side of the urethra, were irregularly shaped structures with a purple, light brown gel-like appearance, composed of lobes which emanated from the base and ran the entire length of the coagulating glands [15,18]. They were made up of many tubules within the main duct of the longitudinal part of the seminal vesicles, and were partially covered by the prostate gland, with tubules running caudally to the urethra and draining into the prostate [18].

The prostate glands were found dorsal to the coagulating glands, and had a similar structure to the seminal vesicles. These paired lobulated structures were creamish-white in color, and had forty to fifty lobes which were slightly spongy, and mostly looped backwards [15]. This description was inconsistent with that of Menezes et al. [18], who described one prostate gland being divided into two equal lobes; a ventral lobe (located laterally to the urethra and divided into a dorsal and a ventral area) and a dorsal lobe (also divided and found on either side of the urethra). 

The paired bulbourethral glands were bean-shaped and located ventro-lateral to the rectum, and dorsal to the pubic symphysis [15]. This disagreed with Menezes et al. [18] who found that the paired bulbourethral glands were located dorso-lateral to the rectum and dorso-cranial to the anal sphincter muscles. The glands were latero-medially compressed, covered dorsally by subcutaneous tissue. They contained one duct that opened into the urethra, before the formation of the penis bulb [15]. 

**Figure 1:**
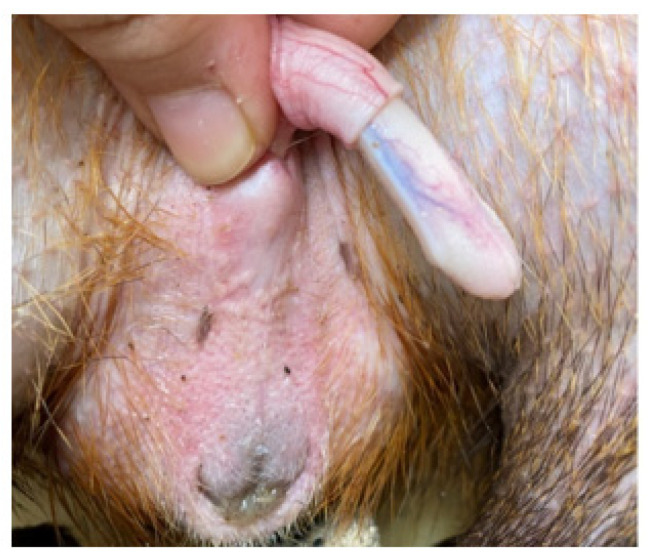
Penis showing lateral cartilage.

**Figure 2:**
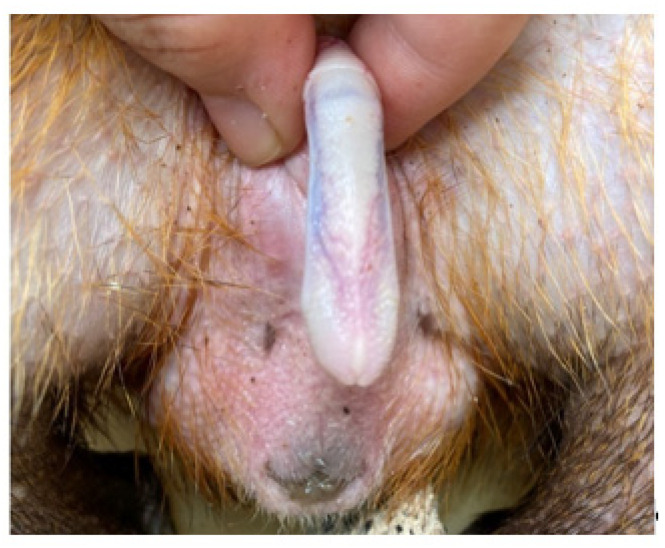
Dorsal view of penis.

**Figure 3:**
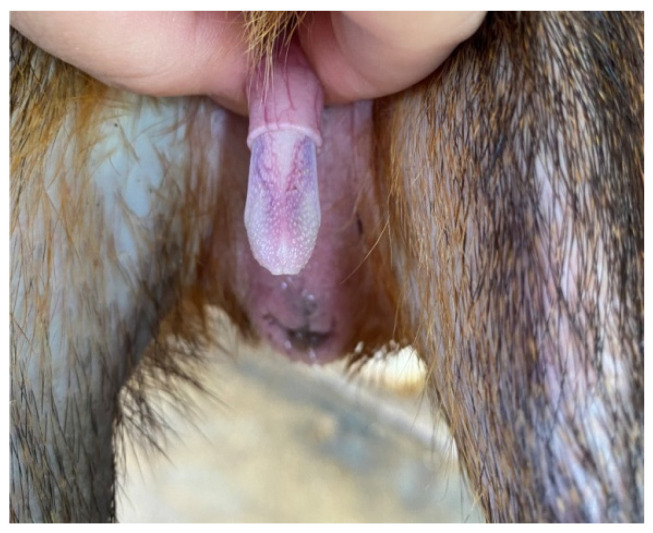
Penile spines visible.

The penis, situated subcutaneously on the ventral side of the pubic symphysis, was cylindrical, laterally compressed, caudally directed and U-shaped [15,17]. The penis was made up of two parts; the glans (glans penis; covered by the prepuce) and the body (corpus penis), which had a distinct junction located just before the bend in the U-shaped penis. The glans penis was covered by penile spines that were raised and directed towards the junction (see Figure 1, Figure 2 and Figure 3). Within the dorsal part of the glans penis was the os penis (2.5 cm in length) [15]. An intromittent sac opened into a slit ventral to the urethral opening, in which two keratinaceous styles were located. The cranial ends of these keratinaceous styles were attached to the caudal end of the intromittent sac and had a mean length of 1.20 ± 0.03 cm. A lateral penile cartilage was found on either side of the glans. The dorsal edge was attached to the lateral side of the glans penis, while its ventral side was free [15]. These descriptions were similar to those of Menezes et al. [17], who also found the presence of the dartos tunic, external and internal spermatic fasciae and cremaster muscle, upon removal of the skin.

Some of these anatomical features worked together in order for erection to take place. Four stages of erection were identified: the penis protruded out of the preputial orifice during stage 1; the lateral penile cartilages were raised vertically and spread laterally during stage 2; stage 3 involved the formation of a penile flower and eversion of the intromittent sac; stage 4 was the completion of erection and ejaculation [19].

### 3.2. Histology of the Male Reproductive Tract

Costa et al. [16] suggested that agoutis were good candidates for increasing their reproductive performance in livestock programs. They had high spermatogenic efficiency and the same seminiferous epithelium cycle pattern and relative stage frequencies as the *Agouti paca*.

Eleven spermatozoa morphologies were reported. The most abundant spermatozoa were normal, while ten had defects, such as a piriform head, micro-cephalic head, retained cytoplasmic droplet, bent neck, double tail, and coiled tail. The spermatozoon was found to have an oval-shaped head, a streamlined mid piece and a thin, tapered tail [20,21]. The lateral penile cartilage consisted of perichondrium, chondroblasts, chondrocytes, intercellular substance, isogenous groups or nest cells, blood vessels, nerves and glands. The peripheral layer consisted of collagenous fibers and fibroblasts, while the second layer (cellular layer) consisted of mesenchymal cells [15]. The corpora cavernosa was made up of erectile tissue covered by a thick layer of dense connective tissue (tunica albuginea). The spongy body of the penis was comprised of erectile tissue around the urethra, lined with endothelium and surrounded by a layer of dense connective tissue (the white tunic of the spongy body) [17].

The seminal vesicles were branched and the mucosa, which was thrown into folds, was lined by a pseudo-stratified columnar epithelium. It consisted of a lamina muscularis mucosa, a tunica muscularis, and a tunica serosa [22]. These findings were also consistent with those of Menezes et al. [18]. There was a significant relationship between the length of the seminal vesicles and the length of the epithelium folds. Table 2 shows the microscopic dimensions of the seminal vesicles.

The mucosa of the coagulating glands was leaf-like and contained tubulo-alveolar glands. The epithelium had apical blebs and cilia on the surface and consisted mainly of pseudo-stratified columnar cells (consistent with the findings of Menezes et al. [18]), with some simple columnar cells. Beneath the epithelium was the lamina propria; however, no distinct lamina muscularis mucosa or tunica submucosa was identifiable. The tunica muscularis contained fibroblasts dispersed between the smooth muscle fibers, and the tunica serosa contained areolar connective tissue in which there were blood vessels [22]. 

The epithelium of the prostate consisted of pseudo-stratified columnar cells and was folded, thus creating a large lumen [18,22]. The mucosa contained tubular and tubulo-alveolar glands. Apart from the epithelial layer, the other layers included the lamina propria, tunica muscularis, and tunica serosa. There was no observable separation between the lamina muscularis mucosa and the tunica submucosa.

The bulbourethral gland consisted of densely packed convoluted tubules with mucous secretory units, and poorly stained cytoplasm and vacuoles [18,22]. The gland was covered by a thick, stratified skeletal tissue layer, and surrounded by a thin capsule of conjunctive tissue [18]. The epithelial lining was made up of simple columnar epithelial cells with oval nuclei, which were located near the basement membrane. These cells were surrounded by a lamina propria [22]. 

Spermatogonial cells (Type A-pale, Type A-dark, intermediate, and Type B) were found in the germinal epithelium of the seminiferous tubules in pre-pubescence, pre-puberty, pubescence and sexually mature agoutis [23,24]. Similar classifications were also used by Assis-Neto et al. [24] in the analysis of spermatogenesis, where they found that birth to five months old was pre-pubescent; six to eight months old was the transition to puberty; puberty occurred at nine to ten months old, and post-puberty from twelve to fourteen months old. 

Some primary spermatocytes were found in the seminiferous cords of pre-pubescent agoutis (in prophase and its sub-phases) [23]. Spermatocytes, in the pachytene phase, were abundant among primary spermatocytes. Sertoli cells were found in all age groups; they exhibited nuclear membrane invaginations and lipid inclusions in the cytoplasm in the pre-pubertal phase. Leydig cells were also discovered in all age groups, but were most abundant in the pubescent and mature groups, and displayed higher metabolic activity during puberty. Spermatozoa were morphologically fully formed at pre-puberty [23]. 

Costa et al. [16] characterized eight stages of the seminiferous epithelium cycle. They found that only one spermatid generation occurred in stage one and the spermatids, which formed several layers in the upper part of the seminiferous epithelium, had round nuclei. Pre-leptotene spermatocytes, pachytene spermatocytes and Type A spermatogonia were also seen. In stage two, the spermatid nuclei started to elongate, and the chromatin was more condensed than previously. Pre-leptotene spermatocytes were transitioning to leptotene and pachytene spermatocytes. Type A spermatogonia were still present. In stage three, the spermatids formed bundles, and the primary spermatocytes were in leptotene and diplotene. Type A spermatogonia were still present. In stage four, meiotic figures of the first and second divisions were observed. Secondary spermatocytes, zygotene and diplotene spermatocytes were also noted. The spermatid bundles were located within Sertoli cells, and Type A spermatogonia were still present. In stage five, two generations of spermatids were seen (newly formed round and elongated spermatids). The spermatid bundles were more packed, and some were located deep within the epithelium. Early pachytene spermatocytes were the predominant cell type located between spermatids and the basal lamina. Type A spermatogonia nuclei were seen. In stage six, the spermatid bundles were close to the seminiferous tubule lumen. Pachytene spermatocyte nuclei were further from the basal lamina and intermediate spermatogonia were seen. Type A spermatogonia were occasionally present. In stage seven the spermatid bundles dissociated, and spermatid nuclei were close to the tubule lumen; small residual bodies were also present. Type B spermatogonia, with round to ovoid nuclei and large amounts of heterochromatin, were seen, while Type A spermatogonia were intermittently found. In addition, also present were pachytene spermatocytes and round and elongated spermatids. In stage eight, the elongated spermatids (with large residual bodies below) were about to be released at the luminal portion of the seminiferous tubule, and pre-leptotene spermatocytes were located close to the basal lamina. 

Costa et al. [16] also reported on the stereology of the Leydig cells: the nuclear diameter was 8.2 ± 0.1 µm; cell volume was 1230 ± 70 µm^3^ (nucleus volume: 280 ± 12 µm^3^; cytoplasm volume: 950 ± 60 µm^3^); cell number per testis was 70 ± 19 × 10^6^; and cell number per gram of testis was 18 ± 3 × 10^6^. In addition, they found that for Sertoli cells, the cell number per testis was 204 ± 30 × 10^6^, and cell number per gram of testis was 57 ± 6 × 10^6^.

The epididymis had a pseudo-stratified, columnar, stereociliated epithelium. Younger, pre-pubescent, agoutis had more clean cells, and older males had more apical cells. Based on morphology, other cell types such as principal, basal and halogen cells were identified. Peri-tubular myoid cells, present in the vas deferens and epididymis, were found to be more abundant after puberty. The vas deferens was composed of muscular layers (two layers in pre-pubescent and pre-pubertal animals and three layers in pubertal and adult animals) and mucosa (pseudo-stratified epithelium) [25].

### 3.3. Reproductive Technologies

Mollineau et al. [26] pioneered the use of electro-ejaculation for collecting spermatozoa from anaesthetized male agoutis, using a lubricated electro-ejaculator probe (12.7 cm long; 1 cm in diameter) inserted 8 cm in the rectum. Anaesthesia was performed using ketamine, intramuscularly, five minutes before electro-ejaculation was performed. However, this protocol was improved by the inclusion of lower dosages of xylazine in combination with ketamine, since xylazine at a dosage of 40 mg/kg resulted in 75% of ejaculate samples containing spermatozoa [27]. To conduct the electro-ejaculation stimuli sequence, six volts were applied for a five second period (on period), followed by a five second rest period (off time). This sequence was repeated using voltages increasing incrementally by one volt, until a maximum of twelve volts was attained. Upon reaching twelve volts, the sequence was repeated until the agouti ejaculated, or until ten minutes had elapsed [26]. This resulted in spermatozoa being present in only 30% of the ejaculate samples. It was concluded that the maximum electro-ejaculation time should be six minutes, with off periods of three to four seconds. The utilized electro-ejaculation technique was able to yield an average spermatozoa concentration of 106.7 ± 31.1 × 10^6^ spermatozoa/mL, while the highest spermatozoa concentration yielded by Mollineau et al. [27] was 431 ± 180 × 10^6^ spermatozoa/mL. 

In contrast, Martinez et al. [28] were able to obtain spermatozoa from all four electro-ejaculated *D. azarae* males. However, a different anesthetic protocol was used whereby they pre-anaesthetized the animals with azaperone (4 mg/kg) and meperidine (4 mg/kg), intramuscularly. Anesthesia was then induced ten minutes later, using xylazine hydrochloride (0.4 mg/kg) and ketamine hydrochloride (20 mg/kg) intramuscularly, followed five minutes later by a lumbosacral application of lidocaine (5 mg/kg). They also utilized another type of electro-ejaculator for wild animals, and the stimuli sequence was different. They conducted a series of four sets of twenty stimuli, starting at two volts, followed by four volts, then six volts and eight volts, with an on period of three seconds and two-minute intervals between each series. Ejaculation (100%) was obtained using six volts, unlike the average ejaculation voltage of 9.33 ± 0.69 V, obtained by Mollineau et al. [26].

Serial and continuous stimuli and electro-ejaculation using ring and longitudinal electrodes, emitting sine waves and square waves, respectively, were evaluated for efficient semen collection [29]. The agoutis were anaesthetized using ketamine (35 mg/kg) and xylazine (5 mg/kg) intramuscularly. It was concluded that ring electrodes with a serial stimuli protocol improved the efficiency of semen collection via electro-ejaculation as 57% of ejaculate samples contained spermatozoa, as compared with 41.33% and 40.8% [27] and 30% [26]. 

Anesthetic protocols that used xylazine (2.5 mg/kg, intramuscularly), ketamine (20 mg/kg, intramuscularly) and a lidocaine hydrochloride lumbosacral epidural (5 mg/kg), or dexmedetomidine (25 µg/kg, intramuscularly) and ketamine (35 mg/kg, intramuscularly), not only facilitated spermatozoa collection, but also provided better analgesia, as reflected by the vital signs recorded [30]. 

Retrograde epididymal washing was another means of recovering spermatozoa [21,31,32,33]. The left testis obtained from castration was utilized [21]. An anesthetic protocol of 5 mg/kg pethidine hydrochloride, intramuscularly, was used as the pre-anesthetic approximately ten minutes before induction, and maintenance was performed using a combination of 35 mg/kg ketamine hydrochloride and 1 mg/kg xylazine hydrochloride, intramuscularly. The epididymal spermatozoa were then collected after separating the testis-epididymis complex and rinsing the cauda epididymis with 0.2 mL of physiological saline (room temperature). The results from this experiment were satisfactory, as viable spermatozoa was collected from all test subjects, with an average spermatozoa concentration of 748 ± 418.66 × 10^6^ spermatozoa/mL [21]. 

Agoutis were euthanized to obtain both testes and a similar procedure, as described above, was used for retrograde epididymal washing [31]. Flushing media (0.5 mL) was used, which was either powdered coconut water (ACP-109c) or Tris extenders. It was found that both solutions were effective, with powdered coconut water (ACP-109c) yielding 300 ± 2 µL and 1.1 ± 0.3 × 10^9^ spermatozoa/mL, and Tris extenders yielding 200 ± 1 µL and 1.1 ± 0.2 × 10^9^ spermatozoa/mL. Similarly, Silva et al. [32] euthanized agoutis and performed retrograde epididymal washing using coconut water (ACP-109c), as described by Silva et al. [31], and yielded 300 ± 2 µL and 1.4 ± 0.3 × 10^9^ spermatozoa/mL. The same method used by Silva et al. [31,32] was later utilized by Castelo et al. [33] in euthanized *D. leporine*, and yielded a volume of 1650 ± 220 µL and 1.04 ± 0.2 × 10^9^ spermatozoa/mL.

Whole cow’s milk at an ultra-high temperature, unpasteurized coconut water, and pasteurized coconut water, were evaluated for their use as semen extenders [34]. The extenders were frozen in plastic bottles (25 mL of extender/bottle), which were subsequently cooled to 5 °C. In the first experiment, the ejaculate samples were diluted with the respective semen extender to 50, 100, 150 and 200 × 10^6^ spermatozoa/mL by slowly adding half of the required volume of semen extender to the ejaculate, and adding the other half twenty minutes later. The samples were filled into 0.25 mL microtubes and refrigerated at 5 °C. The second experiment followed this same method, except for the fact that the sample size per treatment was smaller, and the samples were stored in frozen pellets in liquid nitrogen (−195 °C). Subsequently, samples were thawed in water baths of different temperatures (30, 40, 50, 60 and 70 °C for 20, 30, 40 or 50 s. It was concluded that samples extended with ultra-high temperature whole cow’s milk (100 × 10^6^ spermatozoa/mL) gave the best result in experiment one, as it had the slowest rate of deterioration and the highest means for forward progressive motility % (FPM%) of 59.5 ± 7.75 after 1 day, and 22.0 ± 6.24 after 5 days. For experiment two, the samples that were thawed at 30 °C for 20 s had the highest means for FPM% (12.18 ± 1.33%) and a 85% rate of deterioration [34].

The recovery and cryopreservation of epididymal sperm using powdered coconut water (ACP-109c) and Tris extenders was also performed by other authors [31]. The initial centrifuged samples were extended with either diluent or egg yolk (20%) and stored at 27 °C, and later cooled to 4 °C. Semen was then added to either the powdered coconut water (ACP-109c) or the Tris extenders with egg yolk and 12% glycerol, to result with 6% glycerol in the final extender. The samples were filled into 0.25 mL straws and stored in liquid nitrogen. One week later, after thawing at 37 °C for one minute, it was concluded that powdered coconut water (ACP-109c) was a better extender than Tris extenders, as it had 26.5 ± 2.6% motile sperm with 2.6 ± 0.2 vigor, compared with 9.7 ± 2.6% motile sperm with 1.2 ± 0.3 vigor.

Epididymal sperm could be cryopreserved in either 0.25 mL or 0.50 mL straws. It was recommended that thawing should be done at 37 °C for one minute, as sperm motility was found to be 26.5 ± 2.6% (0.25 mL straw) and 18.4 ± 3% (0.50 mL straw) when thawed at this temperature [32]. Four cryoprotectants (glycerol [3% and 6%], ethylene glycol, dimethylformamide and dimethylsulfoxide [3% and 6%]) were evaluated for their use in the cryopreservation of epididymal sperm [33]. It was concluded that glycerol (3% and 6%) and dimethylsulfoxide (3%) could be used as cryoprotectants, with their post-thawing sperm motility values of 39.5% (glycerol) and 29.5% (dimethylsulfoxide) being better than that achieved by Silva et al. [31].

## 4. Capybara (*Hydrochoerus hydrochaeris*)

### 4.1. Gross Anatomy of the Male Reproductive Tract

Capybaras had no defined scrotum and their testicles were located subcutaneously in the inguinal area [35]. The thick layer of cremaster muscle, together with the separated testicles, allowed for two distinct muscular sacs to be noted. This also allowed for the testicles to retract into the abdomen, especially when stressed. 

There were two testes present, which were found to be positively correlated to the age and body mass (0.14% of the adult body mass). In addition to the mass, the relative volume of interstitial tissue and the proportion of seminiferous tubules with sperm differed between adults and juveniles [36]. Apart from these correlations, age, mass or size of the testis and nasal scent gland [37,38], as well as the proportion of non-spermatogenic tissue in the testis, were positively correlated [38]. There was no correlation between the number of germ cells and testicular volume, or between the number of Leydig cells and testicular volume [39]. However, Costa and Paula [40] disagreed with Herrera [37], as they found no significant correlation between the age and nasal scent gland; rather, they found that serum testosterone concentration was correlated to a proportional increase of the volume of the nasal scent gland and Leydig cell volume.

The average weight of a testicle was 32 g, with small variations within different individual adult males [35]. The testicles had an average length of 4.71 ± 0.80 cm; average width of 2.61 ± 0.55 cm; average thickness of 2.37 ± 0.65 cm; and a calculated volume of 16.97 ± 9.67 cm^3^ [39].

The foreskin was connected to the anus in such a way so as to form a wide anogenital invagination, comprising the penis (flaccid), anus and paranal glands (pair of scent glands). The parallel anal sacs, which were small and circular, were covered with fur and oil secretions. The base of the penis was directed cranially, which then curved ventro-caudally 180°, around the middle third of the penis, so that the distal end faced caudally. They also had a penile bone in the distal third of the free part of the penis. The opening of the penile gland had an inverted ‘T’ shape, which was due to the connection of the external urethral ostium to the opening of the terminal invagination [35].

The accessory sex glands of the capybara included the seminal vesicles and the prostate gland [35,41,42]. Fernandez [41] located the seminal vesicles in the pelvic cavity, dorsal to the urinary bladder and parallel to the vas deferens. They were paired and tubular in shape, with multiple ducts converging into one duct on either side, flowing into the urethra, forming the ejaculatory ostium with the vas deferens. In addition, also in the pelvic cavity, caudal to the seminal vesicles, was the prostate gland, which was described as a paired, multi-lobulated tubular gland. The lobes were enclosed by the tunica serosa, with ducts opening, via two folds, next to the ejaculatory ostium on the lateral surface of the urethra. However, some investigators stated that the prostate was one gland, made up of many lobules, grouped in dorso-medial (smaller lobes) and ventro-lateral regions on each antimere [35]. The vas deferens was described as a tubular organ, found as a continuation of the epididymal tail, parallel to the pampiniform plexus, opening into the urethra and surrounded by the cremaster muscle [41].

### 4.2. Histology of the Male Reproductive Tract

The age of the capybara was found to affect the histology of the testes, as older adults had larger volumes of interstitial tissue, but had seminiferous tubules of smaller diameters, compared with younger capybaras [36]. It was found that the Leydig cell volume density in the testes, inclusive of individual and total volume and Leydig cell number per testis, were high [40,43]. Although there was no significant correlation between testosterone levels and the proportion of Leydig cells, or with Leydig cell numbers per testis, a significant, positive correlation existed between testosterone levels and the individual volume of Leydig cells. It was also noted that the intertubular compartment consisted of numerous Leydig cells (making up 30% of the testicular parenchyma;~0.03% leydigosomatic index), vast lymphatic sinusoids and, to a lesser degree, connective tissue [40].

Capybaras belong to Type III of Fawcett’s classification spectrum, based on the organization of interstitial tissue in the testes being dominated by Leydig cells with few, small, lymphatic blood vessels [36]. However, other authors disagreed, stating that they belonged to Type I of Fawcett’s classification spectrum, based on the distribution of intertubular tissue components (except for the amount of Leydig cells) [44]. 

Histology of the testes confirmed the presence of occasional spermatogonia, primary spermatocytes, spermatids (initial and final), spermatazoon, Sertoli cells and Leydig cells [45,46]. Leydig cells were the most numerous, followed by germ line cells and then Sertoli cells. There was a correlation between the number of germ line cells and Sertoli cells, and a strong negative correlation between the number of germ cells and Leydig cells [45]. The duration of the spermatogenic cycle was fairly long (one cycle lasted 11.9 ± 0.1 days) and consisted of eight stages based on the overall seminiferous epithelium composition, such as the morphology of spermatid nuclei and presence of meiotic divisions [46]. Each stage, 1–8, lasted 1.67, 1.80, 1.87, 1.74, 1.03, 0.83, 1.12 and 1.84 days, respectively. The seminiferous tubules were arranged individually to form tubular bundles, and comprised of a single tunica propria, seminiferous epithelium and lumen [35]. There was approximately 374 m of seminiferous tubules/testicle, and an adult male had an average tubule diameter of 213 µm and an average seminiferous epithelial height of 79.1 µm. 

Numerous tubular structures, communicating with a large central cavity (containing homogenous acidophilic secretions) comprised the secretory part of the seminal vesicles. Pseudo-stratified, columnar epithelium, with some areas of simple columnar epithelium, made up the secretory epithelium. The columnar cells had a basophilic cytoplasm and rounded nucleus (with nucleolus and clear chromatin). The scant amount of basal cells had a small nucleus, clear cytoplasm and intense basophilia. The lamina propria was thin and discrete, and joined the septa of dense connective tissue. The muscular tunic was composed of an inner layer (thin; longitudinal) and an outer layer (thick; circular). The muscular cells had acidophilic cytoplasm and elongated nucleus (with granular chromatin). The tunica adventitia was composed of loose connective tissue, adipose cells and small blood vessels. The prostate gland had similar histological descriptions as the seminal vesicles [41,42].

Rodríguez et al. [47] were the only authors to report on the characteristics of capybaras’ spermatozoa. The average length of the sperm’s head was 5.41 ± 0.7 µm, while its width was 3.77 ± 0.5 µm and the average length of the tail was 27.9 ± 11.3 µm. There were also variations in the shape of the head; diamond-shaped (19.7%), round (7.7%) and elongated (2.7%).

### 4.3. Reproductive Technologies

Rodríguez et al. [47] pioneered the use of electro-ejaculation for collecting spermatozoa from male capybaras. The animals were pre-anaesthetized with atropine (0.04 mg/kg, intramuscularly), followed ten minutes later by sedation with ketamine (5 mg/kg, intramuscularly) and xylazine (0.2 mg/kg, intramuscularly). The trans-rectal probe utilized for the electro-ejaculation was 16 cm long and 2.5 cm in diameter. Following lubrication and insertion of the probe, one volt was applied for four seconds, followed by a rest period of four seconds. This sequence was repeated using a one-volt incremental increase, until six volts were achieved, and the semen was collected in a conical, plastic tube. They were able to obtain samples from all experimental animals, with the highest number of ejaculations collected using six volts. The average volume was 135.5 ± 93.56 µL, with a pH of 8.14 ± 0.38; mass motility of 32.60 ± 13.46%; individual motility of 34 ± 19.81%; viability of 51.3 ± 19.42%; and a sperm concentration of 127 ± 59.01 × 10^6^ spermatozoa/mL.

In addition to electro-ejaculation, Rosenfield et al. [48] also utilized urethral catheterization (after the administration of ketamine and dexmedetomidine) and epididymal aspiration (after hemi-orchiectomy or necropsy) in order to collect semen samples. 

## 5. Paca (*Cuniculus paca*)

### 5.1. Gross Anatomy of the Male Reproductive Tract

The penis had an inverted S-shape [49] and a bony structure along its ventral aspect [50,51]. The glans penis was covered with caudally facing, cornified papillae and had two lateral plates and a pair of corneal spikes protruding from the tip of the glans [50,51]. The upper third portion of the glans also had a transverse groove and another perpendicular to it, with the external urethral ostium located at the intersection of the sulci [51]. The penis had a foreskin, which opened in the pre-putial ostium, whereas the cylindrical body of the penis was composed of the corpus cavernosum, dorsally, and the corpus spongiosum [49].

The pacas had no scrotum and the testicles were located near the inguinal region, within the abdominal cavity. The testicles, which had a yellow-tinged color and were ovoid, were found to be about 7 cm long and 2.5 cm wide [50]. The weight of a testis was 5.9 ± 1.1 g [16]. These descriptions were akin to that of Borges et al. [44], who also described the testis as having a concaved surface where the body of the epididymis ran, while the lateral side was convex. They also noted, upon longitudinal dissection, that there was a thick, white capsule covering it, and that the testicular lobes were formed by septa radiating from the tunica albuginea into the testicular parenchyma, where they then joined to form the testicular mediastinum. Other noted structures of importance included the testicular artery, pampiniform plexus, proximal mesorchium and peritoneum. Stradiotti et al. [51] also noted that the testicles were covered by a thin tunica albuginea, whereas Borges et al. [49] were able to identify the tunica dartos, subcutaneous tissue, external and internal spermatic fascia, vaginal pouch, vaginal cavity and the parietal and visceral layer of the tunica vaginalis, in addition to the tunica albuginea.

The epididymis was seen as a contoured, tubular structure, surrounded by a layer of connective tissue consisting of the typical head, body and tail [49,50,51]. The vas deferens, a straight, tubular structure, began at the tail of the epididymis and ended in the seminal colliculus of the pelvic urethra (therefore no ejaculatory duct was present) [49,50,51].

The accessory sex glands of the paca included the seminal vesicles, prostate gland, coagulating gland, and bulbourethral gland, all of which occurred in pairs [50,52]. Other authors, however, stated that the accessory glands were the ampulla of the vas deferens, seminiferous vesicles, prostate and bulbourethral gland. The ampulla of the vas deferens was described as a dilation in the distal region of the vas deferens [51].

The seminal vesicles were paired white glands, located ventral to the urinary bladder, measuring 4.5 cm in length and 1.7 cm in width. The lobulated edges of the seminal vesicles gave them a branching appearance [50]. Similar gross descriptions were made by other authors, who also noted that they were the largest of the accessory sex glands [51,52]. They made note that these elongated glands had a few digitiform branches, which converged into a main duct in the pelvic cavity. Found on the dorsal surface of the urinary bladder, on either side, they ran parallel to the end of the vas deferens and into the urethra, isolated from the vas deferens, and no ejaculatory duct was present. 

Two coagulating glands were found at the base of the seminal vesicles, with a pair of prostate glands (each divided into two lobes) located caudally [50,52]. Numerous convoluted ducts made up these glands. This was in agreement with Borges et al. [52], who also noted that the elongated coagulating glands had a slightly concave medial surface, and their ducts entered the urethra from along the prostatic ducts and into the prostatic sinus. They also noted that both lobes of the prostate gland had an irregular surface, with the dorsal lobe being darker than the ventral lobe. The dorsal lobe was located dorsal to the pelvic urethra, seminal vesicles and coagulating glands, whereas the ventral lobe was dorso-lateral to the pelvic urethra and coagulating glands. 

The bulbourethral gland was paired, oval, white and located laterally to the middle part of the urethra [50,51,52]. It was compressed latero-medially and covered by a layer of connective tissue and muscle [52]. It had one duct that entered the urethra, cranial to the bulb of the penis, at the point where the pelvic urethra became the penile urethra.

### 5.2. Histology of the Male Reproductive Tract

The penis was evaluated at two locations—the base of the penis and the sacculus urethralis [50]. At the base of the penis, the urethra was found to have a wide lumen, lined with transitional epithelium, with a layer of loose connective tissue and striated muscle below. The corpora cavernosa was not found. In the sacculus urethralis region, an invaginated part of the glans formed a T-shaped duct. In the lumen of this duct, two penile spines were noted, formed by connective tissue surrounded by a stratified epithelium, with a thick keratin layer. The epithelial lining of the sacculus urethralis was also stratified with multiple keratin spines. In addition, also in this cross-section, the outer layer of the penis had a keratinized stratified epithelium, from which spines of this same tissue projected. An os penis was present in the ventral aspect of the penis that showed a pair of spiny structures, formed by stratified epithelium with a thick keratin layer [49,50]. 

The testicle was surrounded by a thin tunica albuginea [50]. Cells at different stages of maturity were found in the numerous seminiferous tubules and the surrounding connective tissue contained few Leydig cells [49,50]. The germinal epithelium of the testes contained Sertoli cells, spermatocytes and rounded spermatids [53,54]. Spermatocytes in diplotene and zygotene and spermatid in differentiation were also observed [54]. Concerning the testis parenchyma, in terms of volume density, 93.4 ± 0.8% was the tubular compartment; 3.2 ± 0.1% was the tunica propria; 84.7 ± 1% was the seminiferous epithelium; 5.5 ± 0.8% was lumen; 6.6 ± 0.8% was the intertubular compartment; 1.6 ± 0.5% was Leydig cells; 1.0 ± 0.1% was blood vessels; and 0.8 ± 0.3% was connective tissue [16]. They also noted that the tubular diameter was 185 ± 5 µm; seminiferous epithelium height was 72 ± 2 µm; tubular length (per g of testis) was 35 ± 2 m; and total tubular length per testis was 172 ± 19 m. They also characterized eight stages of the seminiferous epithelium cycle, which was the same as in the agouti, as mentioned in the relevant section above.

The Leydig cell had a nuclear diameter of 7.2 ± 0.2 µm; a cell volume of 960 ± 130 µm^3^ (nucleus volume: 200 ± 13 µm^3^; cytoplasm volume: 760 ± 120 µm^3^); cell number per testis of 74 ± 34 × 10^6^; and cell number per gram of testis of 17 ± 5 × 10^6^ [16]. In addition, they found that for Sertoli cells, the cell number per testis was 204 ± 13 × 10^6^, and cell number per gram of testis was 43 ± 3 × 10^6^.

The distal end of the epididymis contained striated skeletal muscle [50]. The ducts found in the head, body and initial part of the tail of the epididymis were found to be covered by a pseudo-stratified epithelium, which transitioned to a simple, columnar epithelium as it neared the vas deferens. All three parts of the epididymis also had a thin layer of smooth muscle and connective tissue below the epithelium [49,50,55]. The epididymis had five distinct, but continuous zones [55]. There was a gradual transition among the five zones, which resulted in an overlap of the morphological features between two neighboring zones. The zones were differentiated based on characteristics such as the type and morphology of cells present, presence of spermatozoa, epithelial height and luminal morphology. A pseudo-stratified columnar epithelium, with stereocilia on the apical surface, lined the epididymal duct, with principal, basal, apical and narrow cells comprising the cellular components of the epithelium. The epithelium was also surrounded by the lamina propria and a peri-tubular muscle layer.

The vas deferens was made up of a pseudo-stratified epithelium, with a layer of connective tissue and a thick muscular layer below, comprised of smooth fibers dispersed within the inner and outer layers [50]. Principal and basal cells essentially formed the epithelium [56]. The supranuclear cytoplasm of the principal cells contained numerous lysosomes, adjacent to multi-vesicular bodies with pale or denser content, and endosomes. Vesicles, which differed in shape, size and internal content; coated vesicles; smooth surface vesicles; great vesicles; mitochondria, and rough endoplasmic reticulum, were also seen. Some principal cells, in the supranuclear cytoplasm, contained large lipid inclusions. In the apical cytoplasm of principal cells, caveolae, coated pits, transparent vesicles, multi-vesicular bodies, lysosomes, rough endoplasmic reticulum, polysomes and small dense coated vesicles were seen. There was also an apocrine secretory apparatus protruding into the vas deferens lumen. The basal cells had no contact with the lumen’s surface, and were located next to the basal cytoplasm of principal cells and the basement membrane of the ductus. Basal cells were elongated, with an elongated nucleus, notched nuclear envelope and a scant amount of cytoplasmic organelles. There was a predomination of euchromatin in the nuclear matrix as well [57]. 

The inner surface of the seminal vesicles had a corrugated mucosa consisting of pseudo-stratified and simple columnar epithelium, with connective tissue below. This was in contrast to other authors who stated that the tubules were lined by simple cuboidal epithelium supported by connective and smooth muscle tissue [52]. 

The histology of the two coagulating glands and pair of prostate glands was similar to that of the seminal vesicles, with the only difference being the diameter of the ducts [50]. The mucosa of the coagulating glands consisted of pseudo-stratified and simple columnar epithelium, and the lobes were surrounded by loose connective tissue and smooth muscle fibers [50,52]. Basal cells, with a centrally located nucleus and clear cytoplasm were also noted. The prostatic tubules had an uneven mucosal surface surrounded by thick fibrous muscle tissue, with a simple columnar epithelium that was pseudo-stratified in some areas. It also contained dark cells with an elongated nucleus and dark cytoplasm, clear cells with a round nucleus and clear cytoplasm, and basal cells with an ovoid nucleus and clear cytoplasm. 

Externally, the bulbourethral glands were comprised of striated muscle and dense connective tissue. This connective tissue formed septa which penetrated the glandular parenchyma, creating lobules. The glandular tissue was of the tubulo-alveolar type, with a simple columnar to a simple cuboidal epithelium. Some pyramid-shaped epithelial cells were seen, but the majorities were round in shape, with an ovoid nucleus and clear cytoplasm. The epithelium was supported by loose connective tissue and smooth muscle fibers, which formed the lamina propria [52]. 

The head of the epididymal spermatozoa, which was oval and contained three acrosomal vesicles, measured 7.54 ± 0.82 μm in length and 5.30 ± 0.68 μm in width. The mid-piece measured 5.35 ± 0.83 μm (length) and the tail measured 30.72 ± 2.55 μm (length), giving a total length of 43.87 ± 4.91 μm. In the acrosomal area, vesicular structures under the cytoplasmic membrane were present [58]. Likewise, Ferreira et al. [59] also saw three acrosomal vesicles, and they found that the spermatozoa’s head measured 5.49 ± 0.47 μm in length and 4.02 ± 0.2 μm in width, while the mid-piece measured 6.19 ± 0.73 μm (length) and the tail measured 28.6 ± 2.6 μm (length), giving a total length of 40.86 ± 2.15 μm. These results were also comparable to Cuan-Barrera et al. [60], who found that the spermatozoa’s head measured 5.69 ± 0.18 μm in length, 3.60 ± 0.14 μm in width and 11.32 ± 0.44 μm in area, while the tail measured 24.24 ± 0.40 μm (length), giving a total length of 33.53 ± 0. 42 μm. However, Cuan-Barrera et al. [60] did not report finding acrosomal vesicles. 

### 5.3. Reproductive Technologies

Semen was collected from anaesthetized adults via aspiration. Anesthesia was performed using atropine (0.04 mg/kg) and Zoletil^®^50 (5 mg/kg) in order to perform an orchiectomy, following which the testicles were refrigerated. Afterwards, aspiration of the epididymal tail was performed. Some of the contents of the aspirate were stained with eosin-nigrosin and observed, whereas some samples were diluted with 200 µL of 0.9% NaCl. This allowed for morphometric analysis [60]. 

Stradiotti et al. [61] developed a stimuli protocol for the electro-ejaculation of pacas. This was done by firstly anaesthetizing the animals using acepromazone (1%; 0.1 mg/kg), atropine (0.04 mg/kg), ketamine (20 mg/kg) and xylazine (1.5 mg/kg), all intramuscularly, as well as gauze soaked in saline to cover the eyes. A lubricated probe was inserted about five centimeters into the rectum, while applying slight ventral pressure. Three stimuli series were conducted; the first series consisted of ten stimuli, with one and two volts. The second series consisted of ten stimuli with three and four volts, whereas the third series consisted of ten stimuli with five volts and intervals of two seconds between sets. The ejaculate was collected via a funnel system, into a heated collection tube, wrapped in a protective cover. This resulted in collection of ejaculates with two distinct layers—one more liquid with poor numbers of spermatozoa, and another more viscous with more spermatozoa. Two distinct layers were also reported by Ferreira et al. [59].

Sperm parameters obtained using the floatation method with coconut water powder (ACP 123) were better than those obtained via electro-ejaculation, whereas both ACP 123 and Botusemen special^®^ did not preserve the spermatozoa samples satisfactorily for twenty-four hours [62]. One method used to collect semen was via electro-ejaculation, and this procedure was based on the protocol from Stradiotti et al. [61], with the difference being that atropine was not used. Acepromazine was used as the pre-anesthetic and then five minutes later induction via ketamine and xylazine was performed; the ejaculate was collected via an insulin syringe (without needle), which was then stored in Eppendorf tubes. This resulted in obtaining an average volume of 0.43 ± 0.33 mL, with a mean concentration of 45.5 ± 42.44 × 10^6^ spermatozoa/mL, motility of 33.33 ± 32.14% and mean vigor of 2.6 ± 1.15. 

The second method of semen collection, which was pioneered with respect to pacas, was directly from the tail of the epididymis, using the floatation method, thirty days after electro-ejaculation. The two types of diluents used were coconut water powder (ACP 123) diluted in 50 mL of distilled water, and Botusemen special^®^. After the animals were sacrificed, the testicles were removed, and the epididymis was dissected away. The head of the epididymis was removed, and the remainder was utilized (tail and body). They were immersed in petri dishes containing 1 mL of the respective diluent and transverse cuts were then made along the entire length. After 5 min, 10 µL of the solution was removed and placed on a glass slide for the microscopic evaluation of motility and vigor. To assess the concentration, 10 µL of washed volume was removed and fixed in 1 mL of saline formaldehyde for microscopic evaluation. This resulted in obtaining an average volume of 1.5 mL, with a mean concentration of 197.1 ± 84.9 × 10^6^ spermatozoa/mL, motility of 29.8 ± 34.2% and mean vigor of 2.4 ± 1.9 for the Botusemen special^®^. For the coconut water powder (ACP 123), the average volume and mean concentration were the same, but motility was 63.8 ± 34.2% and mean vigor was 4.2 ± 1.7. It was, however, noted that even without extenders, the epididymal spermatozoa had progressive motility with exceptional vigor and the formation of eddies [62].

Other authors used the flotation method to recover epididymal spermatozoa from the testes of slaughtered males, which resulted in 73% normal spermatozoa, while 27% had defects (6.5% had minor defects and 20.5% had major defects). They used powdered coconut water (ACP-123) and Botusemen special^®^ as the extenders for flotation and found that there were no differences in sperm abnormality rates between both extenders, and both preserved the cell membranes. However, ACP-123 preserved 83.8% of viable spermatozoa compared with the Botusemen special^®^, which preserved 72.9% [57]. 

Unlike Alves [62], Ferreira et al. [59] utilized an artificial vagina to collect spermatozoa and got better results, such as being able to obtain a volume of 2.03 ± 1.51 mL, from the liquid fraction, with a mean sperm motility of 80%. 

## 6. Comparison amongst Hystricomorphic Rodents

The male reproductive system of all three Neo-tropical hystricomorphic rodents contained a penis, paired testes and accessory sex glands. The penises of the agouti and paca were covered in penile spines; they had two keratinized spines in an intromittent sac and two lateral penile cartilages on either side of the glans penis, but no mention of this was made for the capybara. All three did not have defined scrotums, as the testes were found intra-abdominally, mainly in the inguinal region. However, there were variations noted in the accessory sex glands that were present. In the capybara, there were two scent glands, of which there was a positive relationship between testes mass and nasal scent gland volume and size, and they had a unique anogenital invagination and penile gland opening. The paca was unique in that a vaginal pouch and epididymal sinus were present. 

Fawcett’s classification consists of three types, based on the organization of the interstitial tissue in testes [63]. There were differing opinions as to whether capybaras belonged to Type I or Type III of the Fawcett’s classification. Species belonging to Type I tend to have a small amount of Leydig cells, small amounts of connective tissue and extensive peritubular lymphatic sinusoids occupying a large part of the intertubular area. Type II species tend to have clusters of Leydig cells and abundant connective tissue drained by a lymphatic vessel, in each intertubular area. Type III species tend to have abundant Leydig cells that occupy nearly all of the intertubular areas, small amounts of connective tissue and few, small, interstitial lymphatics. However, the agouti and paca were not classified. 

Agoutis and pacas were considered to be medium-sized rodents, weighing between 2.1–2.8 kg and 5.0–9.0 kg, respectively [16,31,57,64]. The capybara, the largest rodent in the world, weighed between 45.0 and 62.0 kg [40,44,46]. All three rodents were considered to be non-seasonal breeders; however, capybaras displayed a peak in births in September [65,66,67]. 

Puberty in male agoutis was attained between 9 and 10 months of age, with pre-puberty from 0 to 5 months, a transitional phase during 6–8 months old, and post-puberty being around 12–14 months old [14]. Puberty in male pacas was achieved slighter older, at 12 months of age [65], and this was even older in male capybaras, at 18 months of age [68]. Agoutis and pacas were said to have high spermatogenic efficiency, thus making them ideal for reproductive programs necessary for conservation and production [16]. However, capybaras were found to have a lower spermatogenic efficiency, but their testes had the highest proportion of Leydig cells to seminiferous tubules seen in mammals [39,40,43,45,46]. 

Agoutis had eight stages of the seminiferous epithelium cycle, characterized based on the morphology of the spermatid nuclei, morphology of the seminiferous epithelium and presence of meiotic division [16]. The spermatogenic cycle lasted 9.5 days, 11.5 days and 11.9 days in the agouti, paca and capybara, respectively [16,46]. The total duration of spermatogenesis was 42.8 days, 51.6 days and 53.6 days in the agouti, paca and capybara, respectively [16,46]. The gonadosomatic index was found to be 0.3%, 0.2% and 0.1% for the agouti, paca and capybara, respectively [16,40,44]. 

Agoutis usually formed monogamous pairs [69] but could also, in captivity, have several females mated with a male without forming an exclusive bond [70]. On the other hand, capybaras were found to be polygynous in nature [48,71], whereas pacas were solitary animals, except for females and their young, with no male–female pairs seen together [71]. Courtship was similar for both agoutis and pacas, a process that usually involved naso-nasal contact (often with vocalizations), following of the female, enurination of the female by the male (causing female agoutis to go into a “frenzy dance”), thumping of the feet, mounting, intromission and finally ejaculation [69,72]. It was noted that male pacas prodded females with the muzzle, but only at night [73]. Capybaras, however, mated in water [70]. A dominant male would follow a female in estrus and sniff her vulva. They would then enter the water and swim for a few minutes, with the female sometimes returning to land before re-entering the water again for copulation.

## 7. Conclusions

The male reproductive system of all three Neo-tropical hystricomorphic rodents showed many similarities regarding the typical male organs—penis, testes and accessory sex glands—with a few differences and unique features. A few different reproductive technologies have been explored in these animals; however, it is clear that more work needs to be performed in order to establish protocols for semen collection, storage and cryopreservation. To the author’s knowledge, artificial insemination, an important and common procedure used in domesticated animals, has not been done in any of these three species. 

## Figures and Tables

**Table 1:** Gross measurements of the male reproductive tract of the agouti (*D. leporina*), according to Mollineau et al. [15].

Component	Mean Length (cm)	Mean Diameter or Width (cm)	Mean Weight (g)
**Testes**	3.67 ± 0.12	1.67 ± 0.04	5.03 ± 0.52
**Vas deferens**	10.98 ± 0.40	0.14 ± 0.01	-
**Seminal vesicles**	4.76 ± 0.15	1.03 ± 0.04	1.28 ± 0.16
**Coagulating glands**	3.10 ± 0.22	1.74 ± 0.12	1.23 ± 0.23
**Prostate glands**	3.50 ± 0.12	1.10 ± 0.02	0.99 ± 0.41
**Bulbourethral glands**	1.47 ± 0.01	1.65 ± 0.11	1.27 ± 0.16
**Penis**	9.90 ± 0.43	1.60 ± 0.17	4.72 ± 0.25
**Glans penis**	3.18 ± 0.22	0.80 ± 0.18	1.80 ± 0.04
**Corpus penis**	6.72 ± 0.43	0.76 ± 0.03	2.94 ± 0.25

**Table 2:** The mean microscopic measurements of some of the components of the male reproductive tract of the agouti (*D. leporina*), according to Mollineau et al. [22].

	Mean Diameter of Lumen (µm)	Mean Width of Tunica Mucosa (µm)	Mean Width of Tunica Muscularis (µm)	Mean Width of Tunica Serosa (µm)
**Seminal vesicle**	883.6 ± 76.83	24.1 ± 0.92	233.1 ± 26.40	38.3 ± 4.26
**Coagulating glands**	488.3 ± 41.96	15.0 ± 1.25	84.5 ± 6.86	24.2 ± 1.84
**Prostate**	995.2 ± 55.70	13.9 ± 1.16	34.2 ± 3.22	39.6 ± 3.73

## Data Availability

Data supporting the results can be found within the manuscript.
